# Effect of Basil Leaves and Wheat Bran Water Extracts on Antioxidant Capacity, Sensory Properties and Microbiological Quality of Shredded Iceberg Lettuce during Storage

**DOI:** 10.3390/antiox9040355

**Published:** 2020-04-24

**Authors:** Małgorzata Sikora, Urszula Złotek, Monika Kordowska-Wiater, Michał Świeca

**Affiliations:** 1 Department of Biochemistry and Food Chemistry, University of Life Sciences, Skromna Str. 8, 20-704 Lublin, Poland; malgorzata.sikora@up.lublin.pl (M.S.); urszula.zlotek@up.lublin.pl (U.Z.); 2 Department of Biotechnology, Microbiology and Human Nutrition, University of Life Sciences in Lublin, 20-704 Lublin, Poland; monika.kordowska-wiater@up.lublin.pl

**Keywords:** iceberg lettuce, unprocessed food, antioxidant capacity, phenolics, potential bioaccessibility, consumer analysis, microbiology, storage, wheat bran, basil

## Abstract

The effect of basil leaf (BLE) and wheat bran (WBE) extracts (potent anti-browning agents), on the phenolic content, antioxidant potential, microbiological quality, and consumer quality of shredded lettuce during storage were studied. Treatment of lettuce with increasing concentrations of BLE proportionally increased the total phenolic content and antioxidant properties. Compared to the control, the treatment enhanced the antiradical properties. This was especially visible during the analysis of the chemical extracts, while this effect was not retained in the potentially bioaccessible fraction. In the lettuce stored for 8 days, the highest reducing potential and ability to quench radicals were observed in samples treated with 1% BLE—33 mg Trolox equivalent/g d.m. and 2.8 mg Trolox equivalent/g d.m., respectively. Compounds exhibiting antiradical properties were easily bioaccessible in vitro. There was no negative effect of the treatments on the consumer quality. Most importantly, after 8 days of storage, lettuce treated with the studied extract, except 10% WBE, had higher microbiological quality. After 8-day storage, the coliforms count was reduced by 84% and 88% in samples treated with 0.5% BLE and 10% WBE, respectively. In conclusion, treatments of shredded lettuce with BLE and WBE maintain or even improve its quality during storage.

## 1. Introduction 

The presence of non-processed fruits and vegetables in daily diet is extremely important for our health because consumption of such food reduces the risk of occurrence of non-communicable diseases including obesity, cardiovascular diseases, diabetes, and some types of cancer e.g., colorectal, breast, and endometrial cancer [1]. According to literature data, almost 11 million deaths worldwide in 2017 were associated with illnesses and disorders caused by improperly balanced diet, including limited access to non-starchy vegetables with a low glycemic index, whole grain cereals, and legumes characterized by high content of phenolic compounds, phytosterols, glucosinolates, dietary fiber, vitamins, and minerals [2]. 

In recent years, an important place on the market of plant-based products has been taken by unprocessed “ready to eat” foods such as mixed lettuce, smoothies, sprouted food, and peeled, washed, and shredded fruits and vegetables. It is a convenient solution for consumers who do not have time for pre-treatment and cooking of raw material but want to eat healthy diets. Moreover, these products are usually characterized by high nutritional quality and pro-health properties [3]. Storage is associated with losses of phenolic compounds and vitamins and undesirable changes in nutrient bioaccessibility and in the color, flavor, and texture of food. During preparation of ready-to-eat lettuce mixes, the leaves are sheered, which increases the activity of some oxidases and promotes growth of endophitic bacteria and molds [4]. Peroxidases and polyphenol oxidases, in the presence of oxygen, catalyze the conversion of phenolic compounds to quinones and lead to the formation of undesirable brown pigments and off-flavored products [5]. Growing microorganisms produced a wide range of metabolites and are able to loosen the structure of food, which supports further undesirable changes and food decay. Importantly, these changes influence negatively the consumer quality of food and are a cause of economic losses. It was proved that reduction of these two factors allows for a significant extension of food self-life [6,7,8]. In light of this, it is extremely important to look for new technologies aimed to effectively preserve post-harvest quality of food during storage.

So far, mainly chemical and physical treatments were employed previously to prevent deterioration of the sensory and nutritional quality of vegetables and fruits. Recently, special attention has been placed on natural extracts that are rich in bioactive compounds (such as polyphenols, organic acids, sulfur compounds) inhibiting enzymatic browning, and improving the nutraceutical potential of food. In our previous study, we have proved that enzymatic browning of shredded iceberg lettuce during cold storage can be effectively limited by application of water infusions of wheat bran and basil leaves. After 8 days of storage, a 91% and 88% reduction of the browning index was recorded after application of 1% basil leaf and 1% wheat bran extracts, respectively [9]. Furthermore, the functional solutions exhibited a mixed mode of POD inhibition and acted as competitive inhibitors of PPO activity. It was proved that phenolics compounds are mainly responsible for the inhibition of the activity of the enzymes. Water extracts of basil contained mainly rosmarinic acid and its sulfone derivative. However, a significant amounts of quercetin glucosides were also detected. The wheat bran extract contained only apigenin derivates (with a dominant apigenin—hexoside-pentoside) without any phenolics acid e.g., ferulic acid [9]. Most importantly, the functional solutions were standardized in terms of phenolics compounds (responsible for inhibitory effect [4]), thus other parts of plants (e.g., stems being waste material) or other sources may be used to reduce the costs. In addition, natural preservatives of food are ecofriendly, cheap, and widely available. They can have a positive impact on the consumer quality of minimally processed food [10]. It has been reported so far that an extract from pine needles of *Cedrus deodara* efficiently inhibited enzymatic darkening of fresh-cut apples [11]. Similarly, Zocca Lomolino & Lante [12] reported that glucosinolates present in processing water left after cooking cabbage leaves displayed an inhibitory effect on PPO activity and an anti-browning effect on grape juice and potato slices. A fruit pulp and core extract from pineapple (*Ananas comosus*), which is a rich source of citric and malic acids, has been demonstrated to be effective in the control of browning of apple slices [13]. The anti-browning effect of the extract is obvious; yet, there were no studies about the influence of functional solutions on the nutritional quality and pro-health properties of food. 

The aim of the current study was to evaluate the impact of functional anti-browning solutions (water extract of basil leaves and wheat bran) on the consumer quality of stored iceberg lettuce. The study is especially focused on changes in the phenolic content, vitamin C, antioxidant capacity, and microbiological quality during cool storage of lettuce. 

## 2. Material and Methods 

### 2.1. Chemicals

l-cysteine, ascorbic acid, ABTS (2,2’-azino-bis(3-ethylbenzothiazoline-6-sulphonic acid), DPPH (2,2-diphenyl-1-picrylhydrazyl), α-amylase (52.7 U/mg), pancreatin (4× UPS), pepsin (541 U/mg), bile extract, and Folin–Ciocalteau phenol reagent were purchased from Sigma–Aldrich company (Poznan, Poland). Nutrient agar, MRS agar, yeast extract, chloramphenicol, VRBL, and other chemicals used for microbiological media were purchased from TL Ltd. (Łódź, Poland). All other chemicals were of analytical grade. Iceberg lettuce (*Lactuca sativa var*.), basil leaves (*Ocimum basilicum* L) and wheat bran from common wheat (*Tricum aestivum* L) were obtained from a local market in Lublin, Poland.

### 2.2. Preparation of a Solution of Inhibitors and Lettuce Treatment

20 mmol/L ascorbic acid and l-cysteine were used as a positive control. Additionally, water infusions of wheat bran (WBE) or basil leaves (BLE) were tested as natural functional anti-browning agents. For preparation of the infusions, 10g of dried samples was mixed with 100 mL of boiling water and left to cool down. Then, the samples were centrifuged (15,000× *g*, 25 °C for 15 min), collected, diluted to working concentrations with water and used for the treatments [9]. 

Iceberg lettuce was cut into squares (2 × 2 cm). Next, the samples (20 g) were soaked for 15 min in 200 mL of distilled water (control sample), 20 mM ascorbic acid (l-AA), 20 mM L–cysteine (LC), 1–10% wheat bran (WBE), and 0.1–0.5% basil leaf (BLE) water extracts. The concentrations used were selected based on significant antibrowning properties (abilities to inhibit activities of polyphenol oxidase and peroxidase) [4].

The excess of solutions was removed using salad spinner (approx. 95% recovery of solution). Then, an excess of the solution was removed. After that, the lettuce was kept in polypropylene boxes at 4 °C for 8 days [9]. The samples were collected for the tests at storage days 3, 5, and 8, rapidly frozen, lyophilized, and kept in polyethylene bags at −20 °C or used immediately for microbiological and consumer quality tests.

### 2.3. Consumer Evaluation

The sensory properties of iceberg lettuce stored for 8 days were evaluated using a five-point method, where the poorest sample received 1 point and the best sample received 5 points. The assessment included the following quality attributes: color, taste, aroma, and texture—where the weighting factor was 0.4, 0.2, 0.2, and 0.2 respectively. The evaluation was conducted by a group of 60 consumers. Coded samples in polypropylene boxes were provided to the panelists for evaluation at 20 °C. 

### 2.4. Low-Molecular Antioxidant Compounds and Antioxidant Properties

#### 2.4.1. Extraction Procedures

##### In Vitro Digestion

In vitro digestion was performed as described previously [14]. For simulated mastication and gastrointestinal digestion, lyophilized lettuce (500 mg of sample) were homogenized in 0.5 mL PBS buffer and 1 mL of simulated salivary fluid [15.1 mmol/L KCl, 3.7 mmol/L KH_2_PO_4_, 13.6 mmol/L NaHCO_3_, 0.15 mmol/L MgCl_2_ (H_2_O)_6_, 0.06 mmol/L (NH_4_)_2_CO_3_, 1.5 mmol/L CaCl_2_, α-amylase (75 U/mL)] and shaken for 10 min at 37 °C. Next, the samples were adjusted to pH 3 with 6 mol/L HCl, suspended in 2 mL of simulated gastric fluid [6.9 mmol/L KCl, 0.9 mmol/L KH_2_PO_4_, 25 mmol/L NaHCO_3_, 47.2 mmol/L NaCl, 0.1 mmol/L MgCl2 (H_2_O)_6_, 0.5 mol/L (NH_4_)_2_CO_3_ 0.15 mmol/L CaCl_2_, pepsin (2000 U/mL)] and shaken for 120 min. at 37 °C. After simulated gastric digestion, the samples were adjusted to pH 7 with 1 M NaOH and suspended in 4 mL simulated intestinal fluid [6.8 mmol/L KCl, 0.8 mmol/L KH_2_PO_4_, 85 mmol/L NaHCO_3_, 38.4 mmol/L NaCl, 0.33 mmol/L MgCl_2_ (H_2_O)_6_, 0.15 mmol/L CaCl_2_, 10 mol/L bile extract, pancreatin (2000 U/mL)]. The prepared samples underwent in vitro intestinal digestion for 120 min. For analysis of the potentially bioaccessible fraction, samples subjected to the gastrointestinal digestion were mixed with an equal volume of methanol to stop the process.

##### Chemical Extraction

For extraction of phenolics, lyophilized samples of lettuce (500 mg of dry mass) were extracted for 1 h at room temperature (300 rpm) in a capped centrifuge tube with 5 mL of different solvents: 50% methanol, 60 mM HCl in 50% methanol, and finally with 60 mM HCl in 70% acetone. The mixture was centrifuged (15 min, 3000× *g*, 22 °C) and the supernatants from all steps were combined.

##### Ascorbic Acid Assay

For ascorbic acid extraction, lyophilized samples of lettuce (500 mg of dry mass) were extracted for 1 h at room temperature (300 rpm) in a capped centrifuge tube with 5 mL of 50% (*w/v*) m-phosphoric acid (MPA). The mixture was centrifuged at 16,000× *g*, and used for further determination.

#### 2.4.2. Low-Molecular Weight Antioxidants

##### Phenolic Content (TPC)

The amount of phenolics samples obtained after chemical extraction and digestion in vitro was determined using the Folin–Ciocalteau reagent [15]. The amount of phenolics is expressed as mg gallic acid equivalents (GAE) per g of dry mass (d.m.). 

##### Ascorbic Acid Content (l-AA)

Total ascorbic acid content was determined as a sum of ascorbic and dehydroascorbic acids according to the methods described earlier by Campos, Ribeiro, Della Lucia, Pinheiro-Sant’Ana & Stringheta [16] with some modification [17]. The samples were analyzed with a Varian ProStar HPLC System separation module (Varian, Palo Alto, California, CA, USA) equipped with a Varian ChromSpher C18 reversed phase column (Si, 5μm, 250 × 4.6 mm) and a ProStar 325 UV–Vis detector. The column thermostat was set at 25 °C. The separation was performed under isocratic elution conditions using a mobile phase consisting of 30 mM KH_2_PO_4_ adjusted with 5 M HCl to pH 3, at a flow-rate of 0.8 mL min^−1^ and detection at 245 nm Quantitative determination was conducted with external standard calculation, using calibration curves of the standard. The ascorbic acid content is expressed in μg per 1 g of dry mass (d.m.).

#### 2.4.3. Antioxidant Properties

##### Ability to Quench ABTS^•+^

2,2′-Azino-bis(3-ethylbenzothiazoline-6-sulfonic acid) diammonium salt (ABTS) was used as a source of free radicals according to Re et al. [18]. The free radical scavenging ability was determined in the samples obtained after chemical extraction and digestion in vitro and is expressed as Trolox equivalents in mg per g of lettuce dry mass (d.m.).

##### Ability to Scavenge DPPH

Free radical scavenging activity was measured using 2,2-Diphenyl-1-picrylhydrazyl radical (DPPH^•^) according to Brand-Williams, Cuvelier, & Berset [19]. The free radical scavenging ability was determined in the samples obtained after chemical extraction and digestion in vitro and is expressed as Trolox equivalents in mg per g of lettuce dry mass (d.m.).

##### Reducing Power (RP)

Reducing power was determined with the method developed by Pulido, Bravo, & Saura-Calixto [20]. Reducing power was determined in the samples obtained after chemical extraction and digestion in vitro and is expressed as Trolox equivalents in mg per g of lettuce dry mass (d.m.).

### 2.5. Microbiological Quality

The following microbiological analyses were performed in accordance with Polish or European standards. For tests, 5 g of lettuce was gently homogenized with 45 mL of Ringer’s solution and shaken for 10 min (60 rpm). 

#### Total Mesophilic Bacteria

The total number of mesophilic bacteria was determined using the plate technique on nutrient agar according to PN EN ISO 4833-2 [21].

#### Lactic Acid Bacteria

The number of lactic acid bacteria was determined with the plate technique on MRS agar according to PN-ISO 15214 [22].

#### Molds and Yeasts

The number of yeasts and molds was determined with the plate technique on agar with glucose, yeast extract, and chloramphenicol according to PN-ISO 21527-1 [23]. Yeast and molds were differentiated according to the morphology of colonies.

#### Coliforms

Coliforms were determined with the plate method on VRBL medium according to PN-ISO 4832 [24].

### 2.6. Theoretical Approach

The following factors were determined for a better understanding of the relationships between biologically active compounds in the light of their bioaccessibility [25]

—the relative phenolic bioaccessibility factor (RBF):

RBF = C_D_/C_CE_

where: C_D_—concentration of compounds after simulated gastrointestinal digestion, C_CE_—concentration of compounds after chemical extraction,

—the relative antioxidant efficiency factor (REF):

REF = A_D_/A_CE_

where: A_D_—activity of the extract after simulated gastrointestinal digestion, A_CE_—activity of the chemical extract.

### 2.7. Statistical Analysis

All experimental results were the mean ± S.D. of three independent experiments (*n* = 9). One-way analysis of variance (ANOVA) and Tukey’s post-hoc test were used to compare the groups (STATISTICA 13, StatSoft, Inc., Tulsa, OK, USA). Differences were considered significant at *p* < 0.05. Microsoft Office Excel was used to perform Pearson’s correlations.

## 3. Results and Discussion

**Figure 1:**
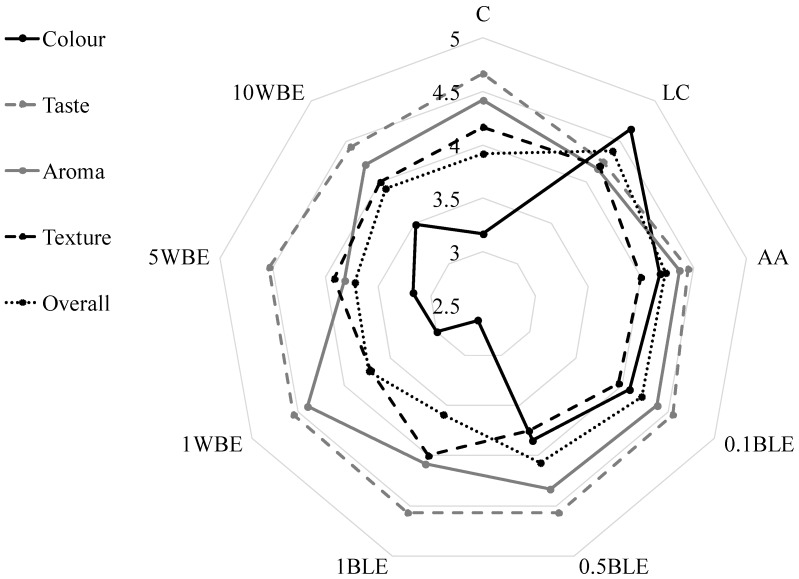
Consumer evaluation of analyzed stored lettuce C-control- lettuce washed in distilled water, LC—lettuce soaked in 20 mM l-cysteine, l-AA—lettuce soaked in 20 mM ascorbic acid, 0.1 BLE, 0.5 BLE; 1BLE—lettuce soaked in 0.1%; 0.5%; 1% water extracts of basil leaves, respectively, 1 WBE, 5 WBE, 10 WBE—storage lettuce soaked in 1%, 5%, 10% water extracts of wheat bran, respectively.

The effect of water extracts decreasing the enzymatic browning of lettuce during storage on the antioxidant potential, low-molecular weight antioxidants, and microbiological quality was estimated. The results of consumer analysis were grouped according to complex sensory properties: color, taste, aroma, and texture (Figure 1). The post-harvest treatment of shredded lettuce with l-cysteine, ascorbic acid, 0.1% and 0.5% BLE, and 10% WBE improved the color of lettuce after 8-day storage (the notes were 4.65, 4.18, 4.08, and 3.85 respectively, which represented a 47%, 32%, 29%, 21%, and 10% increase, respectively, compared to the control). On the other hand, treatment with 1% BLE significantly deteriorated the appearance of the lettuce (score—2.65). The studies showed that the lettuce from all treatments was attractive in terms of taste (score ≥ 4.25) and flavor (score ≥ 4.0). The best score for texture was assigned to the samples soaked in l-cysteine; however, the application of the highest concentrations of WBE and BLE was also effective. The highest overall notes were obtained by the lettuce soaked in l-cysteine, l-ascorbic acid, 0.1% BLE, and 0.5% BLE—4.38, 4.21, 4.24, and 4.07, respectively. 

Since color is the main attribute determining consumer acceptability and describing the freshness of the product, the scores assigned to the treated lettuce were very promising. The quality of low-processed-food is linked with values of the browning index [26]. Capotorto, Amodio, Diaz, de Chiara, & Colelli [27] investigated the effect of different anti-browning solutions such as 0.5% ethanol, 1% ascorbic acid, 0.5% l-cysteine at pH 7, 1% citric acid, and 0.5% 4-hexyloresorcinol on the quality of stored fennel. They reported that dipping in 0.5% ethanol for 2 min was the most effective method for preservation of the visual quality of unprocessed fennel stored for 6 days at 5 °C. Surprisingly, fennel soaked in ascorbic acid and l-cysteine received the worst appearance score than the control sample (water). In turn, Pace, Capotorto, Ventura, & Cefola [28] showed that fresh-cut lettuce soaked in 0.1% l-cysteine was more acceptable by consumers than lettuce soaked in tap water, as it obtained more scores for appearance, texture, absence of browning, and odor. Peng et al. [29] reported that 0.2% and 1% allicin solutions inhibited negative color changes and maintained the visual quality of lettuce without impairing its flavor. In contrast, modified atmosphere packaging of red pigmented “Lollo Rosso” lettuce was found to have a negative impact on the general appearance, texture, and aroma, although improve microbial purity (bacterial counts reduced by c.a. 2 log CFU/g) [30]. 

An important factor affecting the consumer quality of low-processed food is the content of bioactive compounds and the antioxidant potential of products. The highest content of chemically extractable phenolic compounds was determined in lettuce soaked in l-cysteine, ascorbic acid, and 1% WBE and stored for 8 days (Table 1). Compared to the fresh lettuce, an increase by 183%, 103%, and 121%, respectively, was observed. Generally, the highest increase in the content of chemically extractable phenolics (regardless of the day of storage) was recorded in samples treated with l-cysteine and l-ascorbic acid; however, after 8 days of storage, the application of 1% BLE as well as 1% and 5% WBE resulted in a ca. 25%, 50%, and 33% increase, compared to the control. After 3 days of storage, compared to the untreated samples, a slight decrease in the content of phenolics was observed after the treatment with WBE and BLE, except 0.1% BLE. Compared to the fresh lettuce, the process of soaking in l-cysteine, ascorbic acid, 0.1%, 0.5%, and 1% BLE and 1%, 5%, and 10% WBE and further 8-day storage in cold conditions caused an increase in the content of chemically extractable phenolics ranging from 30% (in 0.1% BLE) to 180% (LC). The in vitro digestion released phenolic compounds from both fresh and stored lettuce. The values of TPC in the extracts obtained after digestion of fresh lettuce were higher by 204%, compared to the chemically extractable counterparts. What is important, the phenolics extracted from the lettuce were well bioaccessible in vitro—the RBF values were higher than 1 (Table 4). The highest value of RBF (3.39) was determined for the samples treated with 10% WBE and stored for 3 days. Most importantly, an adverse effect was observed after 5 days of storage for the samples soaked in l-AA, LC, and 1% BLE. 

In the lettuce stored for 8 days, the highest content of phenolics in a potentially bioaccessible fraction was found after the application of WBE; however; the phenolics from the lettuce soaked in BLE were much more bioaccessible in vitro—the RBF values ranged from 1.91 to 2.70.

Generally, the storage decreased the vitamin C content, compared to the fresh lettuce. After the storage, the highest contents of vitamin C (regardless of the duration) were detected in the control samples and those treated with AA and LC (Table 2). However, compared to the untreated counterparts, the application of WBE and BLE caused a significant decrease in the ascorbic acid content. In the samples stored for 8 days, it ranged from 27% to 37% in the samples soaked in 10% WBE and 1% WBE, respectively. Surprisingly, the application of ascorbic acid was not reflected in a subsequent increase of total vitamin C in the stored material. Functional solutions, containing phenolics with reducing properties, should rather protect ascorbic acid against oxidation but in the study, some components of them have a clear, negative influence on ascorbic acid metabolism. It is suggested that they cause a direct oxidation of ascorbic acid and/or inhibit the activity of enzymes responsible for dehydroascorbate conversion e.g., glutathione-depended dehydroascorbate reductase. In the storage conditions (pH c.a. 7) ascorbic acid is unstable and is effectively oxidized to dehydro-l-ascorbic acid, which is highly unstable in an aqueous solution. Dehydroascorbic acid is converted to 2-furoic acid, 3-hydroxy-2-pyrone, 5-methyl-3,4-dihydroxytetrone, and furfural depending on the conditions of the degradation reaction (presence or absence of oxygen) [31]. 

The effect of the anti-browning solutions on the antioxidant capacity of stored iceberg lettuce is presented in Table 3. The low-temperature storage of the lettuce caused an increase in the antioxidant capacity determined in both extraction systems, i.e., in the chemically extractable fractions and the potentially bioaccessible fractions. Compared to the untreated counterparts, all post-harvest treatments significantly improved the ability to quench ABTS cation radical. The highest increase was observed in the l-cysteine-treated samples—2.6, 3.8, and 1.7-fold after 3, 5, and 8 days of storage, respectively. A substantial increase was also determined after the application of WBE and BLE. Compared to the fresh lettuce, the samples soaked in 1% BLE and 10% WBE and stored for 5-days exhibited a 2.09–2.88 increase, respectively. Additionally, after 8 days of storage, except the control samples stored for 3 days and lettuce soaked in 10%WBE, a significant increase in the activity of the potentially bioaccessible fraction was observed. In the subsequent stages of storage, the effect of post-harvest treatments on the activity of the potentially bioaccessible fraction varied and not as clear as in the case of the chemical extracts (Table 3 and Table 4). After 8 days of storage, a 37 % increase was observed in the lettuce treated with 0.5% BLE; however, the application of 10% WBE caused a 21% decrease, compared to the control. The application of the tested functional solution did not have a strong effect on the ability to neutralize DPPH^•^ by the chemically extractable fraction. A slight positive effect was observed in the potentially bioaccessible fraction only in the case of lettuce treated with 0.1% BLE and stored for 3 days as well as lettuce treated with 0.1% BLE and 10% WBE and stored for 8 days. 

After 5 days of storage, the potentially bioaccessible fractions from all the treated samples were characterized by lower ability to neutralize DPPH^•^. Generally, compounds from the stored lettuce exhibiting the ability to quench DPPH^•^ were bioaccessible in vitro (REF values > 1). A negative effect was only observed in the WBE-soaked lettuce after 5 days of storage (Table 4). Importantly, all the studied functional solutions increased the reducing power of stored lettuce. They were more effective than the positive control (ascorbic acid and l-cysteine). After 8 day of storage, the lowest values of reducing power were observed in the samples soaked in ascorbic acid (decrease by 36% compared to the untreated samples). In turn, the highest reducing power was noted in the 0.1BLE, 0.5BLE, 1BLE, and 5WBE lettuce—29.8, 33.2, 29.7, and 34.6 mg TE/g d.m. respectively. Surprisingly, a proportional increase in the RP value, i.e., from 5.58 to 16.2 mg TE/g d.m., was recorded in the negative control (lettuce soaked in distilled water) after 3 and 8 days of storage. A positive effect of the treatment on the activity of the potentially bioaccessible fraction was mainly visible after 3 and 5 days of storage. After 8 days of storage, a significant increase was only recorded in the samples treated with l-cysteine and ascorbic acid, while the application of the function solution caused a slight reduction of the activity. However, except those in the ascorbic acid-treated lettuce stored for 8 days, the compounds exhibiting reducing abilities were poorly bioaccessible in vitro (REF values < 1). 

Storage of fruit and vegetables usually strongly affects low molecules weight antioxidants, which is reflected in their antioxidant potential. Santos, Oliveira, Ibanez, & Herrero [32] showed a significant change (*p* < 0.05) in the total phenolic content in green lettuce after storage (+17.5%). In contrast, Altunkaya & Gökmen [33] observed an opposite relationship during storage of fresh lettuce (*Lactuca sativa*). What is important, the increase in the phenolic content in the lettuce treated with the basil leaf extracts in the present study may also be explained by the fact that the extracts are rich in phenolics, mainly rosmarinic acid and quercetin derivates [9]. Llorach, Martinez-Sánchez, Tomás-Barberán, Gil, & Ferreres [34] reported that the antioxidant activity (DPPH or FRAP) of lettuce was highly correlated with the total phenolic content. This seems to be confirmed by our study, which demonstrated a high correlation between the phenolic content and the ability to neutralize radicals in the chemically extractable fraction obtained from the lettuce stored for 5 and 8 days (*r* = 0.838; *r* = 0.836 respectively). There are sparse studies describing the effect of storage on potentially bioaccessible antioxidants in lettuce; however, the behavior of phenolics during in vitro digestion was studied by Ketnawa, Suwannachot, & Ogawa [35]. They showed that the bioaccessibility of TPC and TFC after digestion was 56–73 and 75–79%, respectively. The analyzed crisphead lettuce exhibited residual activity of ABTS (61–95%) followed by FRAP (70–86%) and DPPH (24–52%). In contrast, Lafarga, Villaró, Rivera, Bobo, & Aguiló-Aguayo [36] demonstrated that in vitro gastrointestinal digestion resulted in increased TPC and antioxidant activities in modern and traditional lettuce varieties. These results are in agreement with those obtained in our study, where the increase in phenolics improved the antioxidant capacity of lettuce. The increase in the content of phenolics observed after in vitro digestion may be caused by release of these components from the food matrix induced by the physicochemical conditions and enzymes. This was previously reported for vegetable-derived beverages (an increase in the TPC by c.a. 15%) [37] or tomatoes and apples (an increase in the TPC from 30.7 and 56.9 to 93.5 and 106.6 mg/100 g, respectively) [38]. 

The studies mentioned above described changes in unprocessed lettuce. However, it should be kept in mind that, during storage of shredded lettuce, the phenolic content and antioxidant activity are tailored by some overlapping processes [39]. In this case, the observed changes are connected with induction of oxidative enzyme activity and phenylopropanoids by wounding. On the one hand, wounding induces the activity of PPO and POD, which consumes phenolics, causes a decrease in their content and antioxidant capacity. The oxidation of phenolic compounds inevitably leads to a drastic decrease in the antioxidant capacity, since the two parameters are positively correlated. An increase in POD and PPO activities was previously recorded during storage of romaine lettuce, where a 3.5- and 2-fold increase in the activity of these enzymes was recorded after 7 days of storage [40]. As shown by Cefola, Pace, Cardinali, D’Antuono, & Serio [41], the activity of POD in fresh-cut iceberg lettuce significantly increased after 16 days of storage (from 1.94 U/g f.w. to 2.66 U/g f.w.). Such an increase was also observed in our studies: a 3- and 2-fold increase in POD and PPO activities was recorded after 5 days of storage [9]. 

On the other hand, wounding may induce de novo synthesis of phenolics. Wounding was found to induce the accumulation of phenolic compounds in unprocessed food (iceberg lettuce) and therefore their antioxidant capacity [41]. In the cited studies, an approx. 2-fold increase in PAL activity and antioxidant capacity was recorded in cut and stored samples. A similar relationship was observed by Zhan et al. [40], who reported that total phenolic content increased in fresh romaine lettuce with the time of storage, which was positively correlated with PAL activity. A significant increase in phenolic metabolism was previously reported for shredded iceberg lettuce as well [35, 37]. In turn, Zhan et al. [40] investigated the influence of continuous varied light exposure on the browning index and quality parameters of fresh-cut romaine lettuce stored for 7 days. 

Both phenolic content and antioxidant activities in stored shredded lettuce can be tailored by modification of storage conditions, which is usually aimed to reduce enzymatic browning. These treatments limit the activity of oxidative enzymes but may induce PAL activity [42]. There are some reports demonstrating an inducing effect of LC and AA (anti-browning agents used for lettuce treatment in our study) on phenylpropanoid metabolism. Altunkaya & Gökmen [33] reported that ascorbic acid and l-cysteine increased the total phenolic content in lettuce, while citric acid and oxalic acid had no effect on TPC. Additionally, they suggested that lettuce phenolic compounds were protected from oxidation by ascorbic acid and l-cysteine. Similarly, Martín-Diana, Rico, & Barry-Ryan [43] evaluated the effectiveness of application of green tea extracts (at concentrations: 0.25, 0.5, 1 g/100mL) to extend the shelf-life of iceberg lettuce stored for 10 days. The solutions preserved vitamin C and carotenoids in the samples and were effective inhibitors of polyphenol oxidase and peroxidase. However, the highest concentration had a negative effect on consumer acceptability (especially the appearance of lettuce). As suggested by the authors, the lowest concentration (0.25 g/100mL) can be applied to minimally processed lettuce. Kim, Kim, Chung, & Moon [44] examined phytoncide from pine as a natural anti-browning agent during storage of fresh-cut lettuce (*Lactuca sativa* cv. Sacramento) for 12 days at 4 °C. They observed that dipping in 1% diluted phytoncide essential oil decreased the activity of enzymes responsible for enzymatic browning. However, these treatments have negative effects on the activity of phenylalanine ammonia lyase and thereby on the total phenolic content. Natural extracts used for improving the consumer quality of shredded lettuce were also studied by Peng et al. [45]. However, they did not study the mechanism of changes, but after 6 days of storage at 4 °C, application of 0.2% and 1% of allicin increased the phenolic content by 78% and 107%, respectively. A similar increase was recorded in our study in lettuce soaked with 1% extracts of basil leaves and wheat bran (increase by 83% and 120%, respectively). A positive impact of phenolic-rich extract form grape seeds on phenolic metabolism was also reported by Altunkaya & Gökmen [46]. They proved that the extract at a concentration of 75 mg/L effectively reduced the degradation of phenolics (inhibition of PPO activity) in fresh-cut lettuce without any negative effect on its organoleptic quality.

It has previously been proved that microbiological contamination is an important determinant of consumer quality of stored shredded lettuce [42,43,44]. The effect of the postharvest treatment on the microbiological quality of the stored iceberg lettuce is presented in Table 5. No molds, yeasts, or lactic bacteria were detected in the stored samples. In all the analyzed samples, an increase in the total count of mesophilic bacteria was observed during the subsequent days of storage. The highest count was found for the lettuce soaked in l-cysteine and stored for 5 and 8 days—6.41 and 7 log_10_ CFU/g f.m., respectively. Compared to the control sample, after 8 days of storage, a significantly lower count of mesophilic bacteria was recorded in the lettuce soaked in the 0.1%, 0.5%, and 1% water extracts of basil leaves (reduction by 45%, 50%, and 55%, respectively) and in the 1% water extract of wheat bran (reduction by 55%). Most importantly, these functional extracts were more effective than ascorbic acid. A very promising effect was found in the case of *Coliforms*. After 8 days of storage, both functional solutions significantly reduced their count. The best results were found in the samples treated with the 0.5% basil leaf and 10% wheat bran extracts, where their count was reduced by 84% and 88%, respectively, compared to the control. 

In turn, as reported by Capotorto et al. [27], citric acid and ascorbic acid have antimicrobial properties because they reduce pH on the surface of fresh-cut products. However, no antimicrobial effect was observed in our study. This may be related to the fact that ascorbic acid exhibits antimicrobial activity at higher concentrations [47]. On the other hand, the increase in the number of mesophilic bacteria in the l-cysteine-treated samples may be explained by the fact that l-cysteine is a good medium for this type of bacteria, as it contains carbon, nitrogen, and other components necessary for growth and development of microorganisms. Peng et al. [45] evaluated the effects of allicin treatment on microbial contamination in fresh-cut lettuce during refrigerated storage (4 °C). Generally, allicin can be an effective tool for improvement of microbial quality of minimally processed food. As shown by our results, the activity of basil leaves extracts and wheat bran was comparable. Ponce, Roura, & Moreira [48] studied the effect of different essential oils on mesophilic, psychotropic, and coliform populations in romaine lettuce leaves. In the case of lettuce stored for 7 days, clove essential oil decreased the coliform count by 16%, while tea tree and rosemary essential oils induced only 8% and 2% reduction, respectively. Similar results were obtained by Skrinjar & Nemet [49], who reported a very strong antimicrobial potential of cinnamon and cloves. Noteworthy, essential oils exert an effect on the organoleptic quality of lettuce. In our study, the water extracts of basil leaves and wheat bran, which are rich sources of phenolic compounds, effectively reduced the growth of coliforms. Previously, Moreno, Scheyer, Romano, & Vojnov [50] found that water extract rich in rosmarinic acid (the main phenolic component of the water extract of basil leaves) effectively inhibited the growth of *E. coli* and *S. aureus*. Also, Nayaka, Londonkar, Umesh, & Tukappa [51] reported that apigenin, i.e., the main phenolic compound in the water extract of wheat bran, exhibited antimicrobial properties. It may be suggested that the antibacterial activity of the extracts can be attributed to the high content of polyphenolic compounds. Adamczak, Ożarowski & Karpiński [52] showed that apigenin and rosmarinic acid were more active against Gram-negative *Escherichia coli* and *Pseudomonas aeruginosa* than Gram-positive *Enterococcus faecalis* and *Staphylococcus aureus* bacteria. Positive effects were visible at concentrations of 500–1000 μg/mL)

## 4. Conclusions

In conclusion, the treatment of shredded iceberg lettuce with natural water extracts from basil leaves and wheat bran improved its quality during storage. 

The application of WBE and BLE had a positive effect on the total phenolic content. However, it caused a significant decrease in the ascorbic acid content. Compared to the fresh lettuce, the ability to quench ABTS cation radicals in the samples stored for 3, 5, and 8 days was the highest after application of 1% WBE, 1% BLE, and 1% BLE, respectively. After eight days of storage, the ability to neutralize DPPH^•^ was the best in lettuce treated with 0.1% BLE and 10% WBE. In turn, the highest reducing power was noted in the samples treated with 0.5% BLE and 5% WBE. The highest overall notes in consumer analysis were obtained for the lettuce soaked in l-cysteine, l-ascorbic acid, and 0.1% BLE, and 0.5% BLE (4.38, 4.21, 4.24, and 4.07, respectively). Generally, the treated lettuce was attractive for consumers, especially in terms of its taste (score ≥ 4.25) and flavor (score ≥ 4.0). Additionally, the functional solutions inhibited the growth of mesophilic bacteria (the most effective were 0.1%, 0.5%, and 1% BLE, and 1% WBE) and coliforms (the most effective were 0.5% BLE and 10% WBE). The final effects were strongly determined by the kind of extract, concentration used for the treatments, and storage time. Compared to the untreated samples stored for eight days, the highest increase of antioxidant potential, without any negative influence on consumer acceptability, was received after application of 0.5% BLE (c.a. 16%).

## Figures and Tables

**Table 1:** Effects of different water extracts on total phenolic content in iceberg lettuce stored by 3, 5, 8 days.

Time of Storage [Days]	Total Polyphenol Content		
	Sample	Chemically Extractable Phenolics	Potentially Bioaccessible Phenolics
0	FL	1.22 ± 0.06 abc	3.71 ± 0.27 efghi
3	C	1.37 ± 0.16 abcd	3.91 ± 0.07 ghi
	LC	2.59 ± 0.15 jkl	3.83 ± 0.04 fghi
	l-AA	2.11 ± 0.01 ghij	3.92 ± 0.05 ghi
	0.1 BLE	1.27 ± 0.05 abc	3.58 ± 0.03 cdefgh
	0.5 BLE	1.30 ± 0.12 abc	2.59 ± 0.67 a
	1 BLE	1.55 ± 0.30 bcdef	3.36 ± 0.32 bcdefg
	1 WBE	1.42 ± 0.04 abcde	3.19 ± 0.09 abcd
	5 WBE	1.32 ± 0.14 abcd	3.04 ± 0.03 abcd
	10 WBE	1.01 ± 0.17 a	3.42 ± 0.27 bcdefg
5	C	1.40 ± 0.22 abcde	2.90 ± 0.02 abc
	LC	2.99 ± 0.41 lm	4.28 ± 0.27 i
	l-AA	1.96 ± 0.18 fghi	3.60 ± 0.07 defgh
	0.1 BLE	1.24 ± 0.06 abc	3.42 ± 0.11 bcdefg
	0.5 BLE	1.40 ± 0.24 abcde	3.80 ± 0.13 fghi
	1 BLE	2.13 ± 0.09 ghij	3.83 ± 0.03 fghi
	1 WBE	1.24 ± 0.03 abc	3.47 ± 0.24 cdefg
	5 WBE	1.43 ± 0.02 abcde	3.79 ± 0.17 fghi
	10 WBE	1.17 ± 0.25 ab	3.66 ± 0.32 defgh
8	C	1.80 ± 0.41 defgh	2.99 ± 0.15 abcd
	LC	3.47 ± 0.05 m	3.88 ± 0.06 ghi
	AA	2.48 ± 0.11 jk	3.29 ± 0.07 bcdefg
	0.1 BLE	1.59 ± 0.02 bcdef	4.13 ± 0.12 hi
	0.5 BLE	1.68 ± 0.15 cdefg	2.76 ± 0.06 ab
	1 BLE	2.25 ± 0.12 hijk	4.28 ± 0.66 i
	1 WBE	2.71 ± 0.07 kl	3.02 ± 0.23 abcd
	5 WBE	2.40 ± 0.19 ijk	3.42 ± 0.27 bcdefg
	10 WBE	1.88 ± 0.26 efgh	3.56 ± 0.38 cdefgh

Means with a different letter (a–l) within the same columns are significantly different. FL—fresh lettuce, C-control—lettuce washed in distilled water, LC—lettuce soaked in 20mM l-cysteine, l-AA—lettuce soaked in 20mM ascorbic acid, 0.1 BLE, 0.5 BLE; 1BLE—lettuce soaked in 0.1%; 0.5%; 1% water extracts of basil leaves, respectively, 1 WBE, 5 WBE, 10 WBE—storage lettuce soaked in 1%, 5%, 10% water extracts of wheat bran, respectively.

**Table 2:** The effect of postharvest treatment on the ascorbic acid content of storage iceberg lettuce.

Time of Storage [Days]	Sample	Total [μg/g d.m.]	Content of Ascorbic Acid [μg/g d.m.]
0	FL	9.06 ± 0.40 efg	8.65 ± 0.23 jk
3	C	9.35 ± 0.05 g	8.75 ± 0.10 k
	LC	8.55 ± 0.02 efg	8.00 ± 0.08 hi
	l-AA	8.36 ± 0.06 efg	7.90 ± 0.34 h
	0.1 BLE	6.60 ± 0.10 d	5.31 ± 0.05 f
	0.5 BLE	6.29 ± 0.21 abcd	5.12 ± 0.11 def
	1 BLE	6.09 ± 0.09 abcd	5.08 ± 0.05 def
	1 WBE	5.45 ± 0.24 abc	4.41 ± 0.18 ab
	5 WBE	5.19 ± 0.05 a	4.17 ± 0.04 a
	10 WBE	5.58 ± 0.18 abcd	4.49 ± 0.08 abc
5	C	9.27 ± 0.12 g	8.48 ± 0.16 ijk
	LC	7.97 ± 0.22 e	7.29 ± 0.17 g
	l-AA	8.76 ± 0.31 efg	8.13 ± 0.08 hij
	0.1 BLE	6.46 ± 0.05 cd	5.26 ± 0.01 ef
	0.5 BLE	6.56 ± 0.01 cd	5.28 ± 0.04 f
	1 BLE	5.97 ± 0.90 abcd	4.93 ± 0.15 bcdef
	1 WBE	5.65 ± 0.50 abcd	4.67 ± 0.06 abcd
	5 WBE	5.58 ± 0.52 ab	4.71 ± 0.12 abcde
	10 WBE	5.33 ± 0.70 ab	4.45 ± 0.10 abc
8	C	8.00 ± 0.23 ef	7.16 ± 0.15 g
	LC	8.81 ± 0.52 efg	7.88 ± 0.33 h
	AA	9.18 ± 0.21 fg	8.31 ± 0.10 hijk
	0.1 BLE	5.84 ± 0.10 abcd	5.23 ± 0.31 def
	0.5 BLE	5.49 ± 0.42 abcd	4.92 ± 0.10 bcdef
	1 BLE	5.59 ± 0.20 abcd	4.50 ± 0.42 abc
	1 WBE	5.32 ± 0.11 ab	4.45 ± 0.22 abc
	5 WBE	6.11 ± 0.35 abcd	4.98 ± 0.20 cdef
	10 WBE	6.37 ± 0.61 bcd	5.06 ± 0.10 def

Means (±SD) with a different letter (a–k) within the same columns are significantly different. FL—fresh lettuce, C-control—lettuce washed in distilled water, LC—lettuce soaked in 20 mM l-cysteine, l-AA—lettuce soaked in 20 mM ascorbic acid, 0.1 BLE, 0.5 BLE; 1BLE—lettuce soaked in 0.1%; 0.5%; 1% water extracts of basil leaves, respectively, 1 WBE, 5 WBE, 10 WBE—storage lettuce soaked in 1%, 5%, 10% water extracts of wheat bran, respectively, d.m.—dry mass.

**Table 3:** Effects of different water extracts on antioxidant activity in iceberg lettuce stored.

Time of Storage [Days]	Sample	Ability to Quench ABTS^•+^ [mg TE/g d.m.]		Ability to Neutralize DPPH^•^ [mg TE/g d.m.]		Reducing Properties [mg TE/g d.m.]	
		CHEM	PBF	CHEM	PBF	CHEM	PBF
0	FL	0.87 ± 0.11 a	1.39 ± 0.03 a	1.16 ± 0.07 ab	1.81 ± 0.15 gh	9.70 ± 0.15 a	5.62 ± 0.13 a
3	C	0.92 ± 0.04 a	1.72 ± 0.03 abc	1.40 ± 0.04 fghi	2.00 ± 0.08 hij	9.78 ± 0.24 a	5.58 ± 0.01 a
	LC	2.44 ± 0.06 jk	2.16 ± 0.09 cdefgh	1.44 ± 0.02 fghijkl	1.67 ± 0.03 efg	28.24 ± 1.03 l	6.44 ± 0.21 b
	l-AA	1.85 ± 0.25 de	1.96 ± 0.13 bcdefg	1.45 ± 0.04 fghijkl	1.81 ± 0.25 gh	28.12 ± 0.27 l	11.33 ± 0.18 f
	0.1BLE	1.57 ± 0.08 bc	1.65 ± 0.03 ab	1.26 ± 0.04 bcde	2.35 ± 0.08 kl	18.38 ± 0.02 c	12.85 ± 0.09 h
	0.5BLE	1.60 ± 0.11 bc	1.91 ± 0.25 bcdef	1.44 ± 0.14 fghijkl	1.34 ± 0.09 a	20.05 ± 0.26 d	12.98 ± 0.34 h
	1 BLE	1.66 ± 0.01 cd	2.03 ± 0.13 bcdefgh	1.41 ± 0.02 fghij	1.54 ± 0.04 abcdef	21.63 ± 0.14 fg	10.78 ± 0.15 e
	1WBE	1.99 ± 0.02 ef	1.77 ± 0.32 abcde	1.41 ± 0.06 fghij	1.39 ± 0.03 abcd	21.52 ± 0.18 fg	9.04 ± 0.32 c
	5WBE	1.42 ± 0.08 b	1.73 ± 0.06 abcde	1.10 ± 0.02 a	1.38 ± 0.06 abcd	16.69 ± 0.21 b	13.48 ± 0.03 i
	10WBE	1.57 ± 0.01 bc	2.39 ± 0.16 ghij	1.54 ± 0.04 jklmn	1.78 ± 0.05 fgh	19.98 ± 1.07 d	19.28 ± 0.25 o
5	C	1.60 ± 0.48 bc	2.19 ± 0.40 defgh	1.35 ± 0.23 defg	2.35 ± 0.03 kl	20.43 ± 0.61 de	10.15 ± 0.02 d
	LC	3.35 ± 0.47 n	2.83 ± 0.42 jkl	1.64 ± 0.24 n	2.25 ± 0.25 jk	27.78 ± 0.68 l	11.55 ± 0.13 f
	l-AA	2.19 ± 0.40 fgh	2.05 ± 0.41 bcdefgh	1.23 ± 0.25 abcd	1.41 ± 0.10 abcd	24.48 ± 0.01 h	16.65 ±0.20 m
	0.1BLE	1.84 ± 0.42 de	2.06 ± 0.42 bcdefgh	1.20 ± 0.25 abc	1.97 ± 0.23 hi	18.94 ± 0.12 c	13.47 ± 0.27 i
	0.5BLE	2.24 ± 0.42 hij	2.73 ± 0.44 ikl	1.32 ± 0.24 cdef	1.65 ± 0.10 defg	26.74 ± 0.72 jk	13.57 ± 0.04 i
	1 BLE	2.51 ± 0.44 k	2.10 ± 0.40 bcdefgh	1.38 ± 0.24 efgh	2.16 ± 0.25 ijk	25.54 ± 0.08 i	15.03 ± 0.16 l
	1WBE	2.02 ± 0.44 efg	2.19 ± 0.30 defgh	1.97 ± 0.25 o	1.75 ± 0.16 fgh	25.08 ± 0.22 hi	12.05 ± 0.49 g
	5WBE	2.03 ± 0.46 efgh	2.40 ± 0.27 ghij	1.47 ± 0.06 ghijkl	1.35 ± 0.08 ab	21.08 ± 0.46 ef	13.67 ± 0.15 i
	10WBE	1.82 ± 0.48 de	2.71 ± 0.21 ijkl	1.44 ± 0.01 fghijkl	1.33 ± 0.04 a	22.14 ± 0.26 g	18.53 ± 0.16 n
8	C	2.22 ± 0.13 ghi	2.31 ± 0.02 fghi	1.56 ± 0.10 lmn	1.83 ± 0.08 gh	28.09 ± 0.16 l	16.2 ± 0.15 m
	LC	3.76 ± 0.22 o	2.89 ± 0.21 kl	1.42 ± 0.02 fghijk	2.00 ± 0.03 hij	27.61 ± 0.06 kl	18.15 ± 0.02 n
	l-AA	2.80 ± 0.06 lm	2.15 ± 0.20 cdefgh	1.38 ± 0.06 efgh	1.38 ± 0.36 abc	18.02 ± 0.16 c	23.6 ± 0.27 p
	0.1BLE	2.43 ± 0.22 ijk	2.47 ± 0.47 hijk	1.40 ± 0.10 fghi	2.18 ± 0.08 ijk	29.76 ± 0.61 m	14.3 ± 0.08 j
	0.5BLE	2.78 ± 0.01 lm	3.17 ± 0.20 i	1.61 ± 0.12 mn	1.43 ± 0.05 abcde	33.21 ± 0. 05 n	16.6 ± 0.03 m
	1 BLE	2.86 ± 0.22 m	2.23 ± 0.12 efgh	1.36 ± 0.02 defg	1.63 ± 0.04 cdefg	29.70 ± 0.33 m	14.9 ± 0.23 kl
	1WBE	2.59 ± 0.10 kl	2.40 ± 0.03 ghij	1.52 ± 0.05 ijklmn	1.60 ± 0.02 bcefg	26.58 ± 0.23 j	12.4 ± 0.42 g
	5WBE	2.58 ± 0.01 kl	2.38 ± 0.18 ghij	1.55 ± 0.14 klmn	1.31 ± 0.03 a	34.56 ± 0.76 o	14.5 ± 0.02 jk
	10WBE	2.42 ± 0.02 ijk	1.82 ± 0.03 abcde	1.51 ± 0.03 hijklm	2.52 ± 0.25 l	24.65 ± 0.95 hi	14.6 ± 0.42 jkl

Means (±SD) with different letter (a–n) within the same columns are significantly different. FL—fresh lettuce, C-control—lettuce washed in distilled water, LC—lettuce soaked in 20 mM l-cysteine, l-AA—lettuce soaked in 20 mM ascorbic acid, 0.1 BLE, 0.5 BLE; 1 BLE—lettuce soaked in 0.1%; 0.5%; 1% water extracts of basil leaves, respectively, 1 WBE, 5 WBE, 10 WBE—storage lettuce soaked in 1%, 5%, 10% water extracts of wheat bran, respectively, TE—Trolox equivalent, d.m.—dry mass, CHEM-chemically extractable phenolics, PBF—potentially bioaccessible phenolics.

**Table 4:** The relative phenolic bioaccessibility and antioxidant efficiency factor.

Time of Storage [Days]	Sample	RBF	REF_abts_	REF_dpph_	REF_rp_
	FL	3.04	1.60	1.55	0.58
3	C	2.86	1.87	1.43	0.57
	LC	1.48	0.89	1.16	0.23
	l-AA	1.86	1.06	1.25	0.40
	0.1 BLE	2.82	1.05	1.87	0.70
	0.5 BLE	2.00	1.19	0.93	0.65
	1 BLE	2.16	1.23	1.09	0.50
	1 WBE	2.24	1.04	1.03	0.42
	5 WBE	2.31	1.22	1.25	0.81
	10 WBE	3.39	1.52	1.16	0.96
5	C	2.07	1.37	1.74	0.50
	LC	1.43	0.85	1.37	0.42
	l-AA	1.84	0.94	1.14	0.68
	0.1 BLE	2.75	1.12	1.65	0.71
	0.5 BLE	2.70	1.22	1.25	0.51
	1 BLE	1.80	0.84	1.57	0.59
	1 WBE	2.79	1.09	0.88	0.48
	5 WBE	2.65	1.18	0.92	0.65
	10 WBE	3.13	1.49	0.92	0.84
8	C	1.66	1.04	1.35	0.58
	LC	1.12	0.77	1.45	0.66
	l-AA	1.33	0.77	0.99	1.31
	0.1 BLE	2.59	1.02	1.54	0.48
	0.5 BLE	2.70	1.14	1.11	0.50
	1 BLE	1.91	0.78	0.95	0.50
	1 WBE	1.11	0.93	1.03	0.47
	5 WBE	1.42	0.92	0.84	0.42
	10 WBE	1.89	0.75	1.56	0.59

FL—fresh lettuce, C-control—lettuce washed in distilled water, LC—lettuce soaked in 20 mM l-cysteine, l-AA—lettuce soaked in 20 mM ascorbic acid, 0.1 BLE, 0.5 BLE; 1 BLE—lettuce soaked in 0.1%; 0.5%; 1% water extracts of basil leaves, respectively, 1 WBE, 5 WBE, 10 WBE—storage lettuce soaked in 1%, 5%, 10% water extracts of wheat bran, respectively, RBF—the relative phenolic bioaccessibility factor, REF—the relative antioxidant efficiency factor.

**Table 5:** The effect of postharvest treatment on the microbiological quality of storage iceberg lettuce.

Time of Storage [Days]	Count of Microorganisms [Log_10_ CFU/g f.m.]				
	Sample	Total Mesophilic Bacteria	*Coliforms*	Molds and Yeasts	Lactic bacteria
0	FL	4.43 a	1.91 ab	n.d.	n.d.
5	C	5.59 b	2.51 ef	n.d.	n.d.
	LC	6.41 f	3.42 i	n.d.	n.d.
	l-AA	5.63 b	1.98 ab	n.d.	n.d.
	0.1 BLE	5.44 ab	1.70 a	n.d.	n.d.
	0.5 BLE	5.44 ab	2.33 bcde	n.d.	n.d.
	1 BLE	5.63 b	2.59 fg	n.d.	n.d.
	1 WBE	5.55 b	2.44 def	n.d.	n.d.
	5 WBE	5.64 b	2.41 cdef	n.d.	n.d.
	10 WBE	6.04 c	2.41 cdef	n.d.	n.d.
8	C	6.38 f	2.67 g	n.d.	n.d.
	LC	7.00 h	3.33 h	n.d.	n.d.
	AA	6.22 d	2.71 g	n.d.	n.d.
	0.1 BLE	6.13 c	2.13 abc	n.d.	n.d.
	0.5 BLE	6.09 c	1.86 a	n.d.	n.d.
	1 BLE	6.04 c	2.15 abcd	n.d.	n.d.
	1 WBE	6.04 c	2.07 abc	n.d.	n.d.
	5 WBE	6.33 e	2.16 abcd	n.d.	n.d.
	10 WBE	6.77 g	1.74 a	n.d.	n.d.

Means with different letter (a–i) within the same columns are significantly different. FL—fresh lettuce, C-control—lettuce washed in distilled water, LC—lettuce soaked in 20 mM l-cysteine, l-AA—lettuce soaked in 20 mM ascorbic acid, 0.1 BLE, 0.5 BLE; 1BLE—lettuce soaked in 0.1%; 0.5%; 1% water extracts of basil leaves, respectively, 1 WBE, 5 WBE, 10 WBE—storage lettuce soaked in 1%, 5%, 10% water extracts of wheat bran, respectively, CFU—colony-forming unit, f.m.—fresh mass, n.d.—not detected.
